# Ten simple rules for responsible big data research

**DOI:** 10.1371/journal.pcbi.1005399

**Published:** 2017-03-30

**Authors:** Matthew Zook, Solon Barocas, danah boyd, Kate Crawford, Emily Keller, Seeta Peña Gangadharan, Alyssa Goodman, Rachelle Hollander, Barbara A. Koenig, Jacob Metcalf, Arvind Narayanan, Alondra Nelson, Frank Pasquale

**Affiliations:** 1 Department of Geography, University of Kentucky, Lexington, Kentucky, United States of America; 2 Microsoft Research, New York, New York, United States of America; 3 Data & Society, New York, New York, United States of America; 4 Information Law Institute, New York University, New York, New York, United States of America; 5 Department of Media and Communications, London School of Economics, London, United Kingdom; 6 Harvard-Smithsonian Center for Astrophysics, Harvard University, Cambridge, Massachusetts, United States of America; 7 Center for Engineering Ethics and Society, National Academy of Engineering, Washington, DC, United States of America; 8 Institute for Health Aging, University of California-San Francisco, San Francisco, California, United States of America; 9 Ethical Resolve, Santa Cruz, California, United States of America; 10 Department of Computer Science, Princeton University, Princeton, New Jersey, United States of America; 11 Department of Sociology, Columbia University, New York, New York, United States of America; 12 Carey School of Law, University of Maryland, Baltimore, Maryland, United States of America; Whitehead Institute, UNITED STATES

## Introduction

The use of big data research methods has grown tremendously over the past five years in both academia and industry. As the size and complexity of available datasets has grown, so too have the ethical questions raised by big data research. These questions become increasingly urgent as data and research agendas move well beyond those typical of the computational and natural sciences, to more directly address sensitive aspects of human behavior, interaction, and health. The tools of big data research are increasingly woven into our daily lives, including mining digital medical records for scientific and economic insights, mapping relationships via social media, capturing individuals’ speech and action via sensors, tracking movement across space, shaping police and security policy via “predictive policing,” and much more.

The beneficial possibilities for big data in science and industry are tempered by new challenges facing researchers that often lie outside their training and comfort zone. Social scientists now grapple with data structures and cloud computing, while computer scientists must contend with human subject protocols and institutional review boards (IRBs). While the connection between individual datum and actual human beings can appear quite abstract, the scope, scale, and complexity of many forms of big data creates a rich ecosystem in which human participants and their communities are deeply embedded and susceptible to harm. This complexity challenges any normative set of rules and makes devising universal guidelines difficult.

Nevertheless, the need for direction in responsible big data research is evident, and this article provides a set of “ten simple rules” for addressing the complex ethical issues that will inevitably arise. Modeled on *PLOS Computational Biology*’s ongoing collection of rules, the recommendations we outline involve more nuance than the words “simple” and “rules” suggest. This nuance is inevitably tied to our paper’s starting premise: all big data research on social, medical, psychological, and economic phenomena engages with human subjects, and researchers have the ethical responsibility to minimize potential harm.

The variety in data sources, research topics, and methodological approaches in big data belies a one-size-fits-all checklist; as a result, these rules are less specific than some might hope. Rather, we exhort researchers to recognize the human participants and complex systems contained within their data and make grappling with ethical questions part of their standard workflow. Towards this end, we structure the first five rules around how to reduce the chance of harm resulting from big data research practices; the second five rules focus on ways researchers can contribute to building best practices that fit their disciplinary and methodological approaches. At the core of these rules, we challenge big data researchers who consider their data disentangled from the ability to harm to reexamine their assumptions. The examples in this paper show how often even seemingly innocuous and anonymized data have produced unanticipated ethical questions and detrimental impacts.

This paper is a result of a two-year National Science Foundation (NSF)-funded project that established the Council for Big Data, Ethics, and Society, a group of 20 scholars from a wide range of social, natural, and computational sciences (http://bdes.datasociety.net/). The Council was charged with providing guidance to the NSF on how to best encourage ethical practices in scientific and engineering research, utilizing big data research methods and infrastructures [1].

## 1. Acknowledge that data are people and can do harm

One of the most fundamental rules of responsible big data research is the steadfast recognition that most data represent or impact people. Simply starting with the assumption that all data are people until proven otherwise places the difficulty of disassociating data from specific individuals front and center. This logic is readily evident for “risky” datasets, e.g., social media with inflammatory language, but even seemingly benign data can contain sensitive and private information, e.g., it is possible to extract data on the exact heart rates of people from YouTube videos [2]. Even data that seemingly have nothing to do with people might impact individuals’ lives in unexpected ways, e.g., oceanographic data that change the risk profiles of communities’ and properties’ values or Exchangeable Image Format (EXIF) records from photos that contain location coordinates and reveal the photographer’s movement or even home location.

Harm can also result when seemingly innocuous datasets about population-wide effects are used to shape the lives of individuals or stigmatize groups, often without procedural recourse [3,4]. For example, social network maps for services such as Twitter can determine credit-worthiness [5], opaque recidivism scores can shape criminal justice decisions in a racially disparate manner [6], and categorization based on zip codes resulted in less access to Amazon Prime same-day delivery service for African-Americans in United States cities [7]. These high-profile cases show that apparently neutral data can yield discriminatory outcomes, thereby compounding social inequities.

Other cases show that “public” datasets are easily adapted for highly invasive research by incorporating other data, such as Hague et al.’s [8] use of property records and geographic profiling techniques to allegedly identify the pseudonymous artist Banksy [9]. In particular, data ungoverned by substantive consent practices, whether social media or the residual DNA we continually leave behind us, may seem public but can cause unintentional breaches of privacy and other harms [9,10].

Start with the assumption that data are people (until proven otherwise), and use it to guide your analysis. No one gets an automatic pass on ethics.

## 2. Recognize that privacy is more than a binary value

Breaches of privacy are key means by which big data research can do harm, and it is important to recognize that privacy is contextual [11] and situational [12], not reducible to a simple public/private binary. Just because something has been shared publicly does not mean any subsequent use would be unproblematic. Looking at a single Instagram photo by an individual has different ethical implications than looking at someone’s full history of all social media posts. Privacy depends on the nature of the data, the context in which they were created and obtained, and the expectations and norms of those who are affected. Understand that your attitude towards acceptable use and privacy may not correspond with those whose data you are using, as privacy preferences differ across and within societies.

For example, Tene and Polonetsky [13] explore how pushing past social norms, particularly in novel situations created by new technologies, is perceived by individuals as “creepy” even when they do not violate data protection regulations or privacy laws. Social media apps that utilize users’ locations to push information, corporate tracking of individuals’ social media and private communications to gain customer intelligence, and marketing based on search patterns have been perceived by some to be “creepy” or even outright breaches of privacy. Likewise, distributing health records is a necessary part of receiving health care, but this same sharing brings new ethical concerns when it goes beyond providers to marketers.

Privacy also goes beyond single individuals and extends to groups [10]. This is particularly resonant for communities who have been on the receiving end of discriminatory data-driven policies historically, such as the practice of redlining [14, 15]. Other examples include community maps—made to identify problematic properties or an assertion of land rights—being reused by others to identify opportunities for redevelopment or exploitation [16]. Thus, reusing a seemingly public dataset could run counter to the original privacy intents of those who created it and raise questions about whether it represents responsible big data research.

Situate and contextualize your data to anticipate privacy breaches and minimize harm. The availability or perceived publicness of data does not guarantee lack of harm, nor does it mean that data creators consent to researchers using their data.

## 3. Guard against the reidentification of your data

It is problematic to assume that data cannot be reidentified. There are numerous examples of researchers with good intentions and seemingly good methods failing to anonymize data sufficiently to prevent the later identification of specific individuals [17]; in other cases, these efforts were extremely superficial [18, 19]. When datasets thought to be anonymized are combined with other variables, it may result in unexpected reidentification, much like a chemical reaction resulting from the addition of a final ingredient.

While the identificatory power of birthdate, gender, and zip code is well known [20], there are a number of other parameters—particularly the metadata associated with digital activity—that may be as or even more useful for identifying individuals [21]. Surprising to many, unlabeled network graphs—such as location and movement, DNA profiles, call records from mobile phone data, and even high-resolution satellite images of the earth—can be used to reidentify people [22]. More important than specifying the variables that allow for reidentification, however, is the realization that it is difficult to recognize these vulnerable points a priori [23]. Factors discounted today as irrelevant or inherently harmless—such as battery usage—may very well prove to be a significant vector of personal identification tomorrow [24]. For example, the addition of spatial location can turn social media posts into a means of identifying home location [25], and Google’s reverse image search can connect previously separate personal activities—such as dating and professional profiles—in unanticipated ways [26]. Even data about groups—“aggregate statistics”—can have serious implications if they reveal that certain communities, for example, suffer from stigmatized diseases or social behavior much more than others [27].

Identify possible vectors of reidentification in your data. Work to minimize them in your published results to the greatest extent possible.

## 4. Practice ethical data sharing

For some projects, sharing data is an expectation of the human participants involved and thus a key part of ethical research. For example, in rare genetic disease research, biological samples are shared in the hope of finding cures, making dissemination a condition of participation. In other projects, questions of the larger public good—an admittedly difficult to define category—provide compelling arguments for sharing data, e.g., the NIH-sponsored database of Genotypes and Phenotypes (dbGaP), which makes deidentified genomic data widely available to researchers, democratizing access, or the justice claim made by the Institute of Medicine about the value of mandating that individual-level data from clinical trials be shared among researchers [28]. Asking participants for broad, as opposed to narrowly structured consent for downstream data management makes it easier to share data. Careful research design and guidance from IRBs can help clarify consent processes. However, we caution that even when broad consent was obtained upfront, researchers should consider the best interests of the human participant, proactively considering the likelihood of privacy breaches and reidentification issues. This is of particular concern for human DNA data, which is uniquely identifiable.

These types of projects, however—in which rules of use and sharing are well governed by informed consent and right of withdrawal—are increasingly the exception rather than the rule for big data. In our digital society, we are followed by data clouds composed of the trace elements of daily life—credit card transactions, medical test results, closed-circuit television (CCTV) images and video, smart phone apps, etc.—collected under mandatory terms of service rather than responsible research design overseen by university compliance officers. While we might wish to have the standards of informed consent and right of withdrawal, these informal big data sources are gathered by agents other than the researcher—private software companies, state agencies, and telecommunications firms. These data are only accessible to researchers after their creation, making it impossible to gain informed consent a priori, and contacting the human participants retroactively for permission is often forbidden by the owner of the data or is impossible to do at scale.

Of course, researchers within software companies and state institutions collecting these data have a special responsibility to address the terms under which data are collected; but that does not exempt the end-user of shared data. In short, the burden of ethical use (see Rules 1 to 3) and sharing is placed on the researcher, since the terms of service under which the human subjects’ data were produced can often be extremely broad with little protection for breaches of privacy. In these circumstances, researchers must balance the requirements from funding agencies to share data [29] with their responsibilities to the human beings behind the data they acquired. A researcher needs to inform funding agencies about possible ethical concerns before the research begins and guard against reidentification before sharing.

Share data as specified in research protocols, but proactively address concerns of potential harm from informally collected big data.

## 5. Consider the strengths and limitations of your data; big does not automatically mean better

In order to do both accurate and responsible big data research, it is important to ground datasets in their proper context including conflicts of interests. Context also affects every stage of research: from data acquisition, to cleaning, to interpretation of findings, and dissemination of the results. During the step of data acquisition, it is crucial to understand both the source of the data and the rules and regulations with which they were gathered. This is especially important in cases of research conducted in relatively loose regulatory environments, in which use of answers to research questions may conflict with the expectations of those who provided the data. One possible approach might be the ethical norms employed to track the provenance of artifacts, often in cooperation and collaboration with the communities from which they come (e.g., archaeologists working in indigenous communities to determine the disposition of material culture). In a similar manner, computer scientists use data lineage techniques to track the evolution of a dataset and often to trace bugs in the data.

Being mindful of the data’s context provides the foundation for clarifying when your data and analysis are working and when they are not. While it is tempting to interpret findings based on big data as a clear outcome, a key step within scientific research is clearly articulating what data or an indicator represent and what they do not. Are your findings as clear-cut if your interpretation of a social media posting switches from a recording of fact to the performance of a social identity? Given the messy, almost organic nature of many datasets derived from social actions, it is fundamental that researchers be sensitive to the potential multiple meanings of data.

For example, is a Facebook post or an Instagram photo best interpreted as an approval/disapproval of a phenomenon, a simple observation, or an effort to improve status within a friend network? While any of these interpretations are potentially valid, the lack of context makes it even more difficult to justify the choice of one understanding over another. Reflecting on the potential multiple meanings of data fosters greater clarity in research hypotheses and also makes researchers aware of the other potential uses of their data. Again, the act of interpretation is a human process, and because the judgments of those (re)using your data may differ from your own, it is essential to clarify both the strengths and shortcomings of the data.

Document the provenance and evolution of your data. Do not overstate clarity; acknowledge messiness and multiple meanings.

## 6. Debate the tough, ethical choices

Research involving human participants at federally funded institutions is governed by IRBs charged with preventing harm through well-established procedures and are familiar to many researchers. IRBs, however, are not the sole arbiter of ethics; many ethical issues involving big data are outside of their governance mandate. Precisely because big data researchers often encounter situations that are foreign to or outside of the mandate of IRBs, we emphasize the importance of debating the issues within groups of peers.

Rather than a bug, the lack of clear-cut solutions and governance protocols should be more appropriately understood as a feature that researchers should embrace within their own work. Discussion and debate of ethical issues is an essential part of professional development—both within and between disciplines—as it can establish a mature community of responsible practitioners. Bringing these debates into coursework and training can produce peer reviewers who are particularly well placed to raise these ethical questions and spur recognition of the need for these conversations.

A precondition of any formal ethics rules or regulations is the **capacity** to have such open-ended debates. As digital social scientist and ethicist Annette Markham [30] writes, “we can make [data ethics] an easier topic to broach by addressing ethics as being about choices we make at critical junctures; choices that will invariably have impact.” Given the nature of big data, bringing technical, scientific, social, and humanistic researchers together on projects enables this debate to emerge as a strength because, if done well, it provides the means to understand the ethical issues from a range of perspectives and disrupt the silos of disciplines [31]. There are a number of good models for interdisciplinary ethics research, such as the trainings offered by the Science and Justice research center at the University of California, Santa Cruz [32] and Values in Design curricula [33]. Research ethics consultation services, available at some universities as a result of the Clinical and Translational Science Award (CTSA) program of the National Institutes of Health (NIH), can also be resources for researchers [34].

Some of the better-known “big data” ethical cases—i.e., the Facebook emotional contagion study [35]—provide extremely productive venues for cross-disciplinary discussions. Why might one set of scholars see this as a relatively benign approach while other groups see significant ethical shortcomings? Where do researchers differ in drawing the line between responsible and irresponsible research and why? Understanding the different ways people discuss these challenges and processes provides an important check for researchers, especially if they come from disciplines not focused on human subject concerns.

Moreover, the high visibility surrounding these events means that (for better or worse) they represent the “public” view of big data research, and becoming an active member of this conversation ensures that researchers can give voice to their insights rather than simply being at the receiving end of policy decisions. In an effort to help these debates along, the Council for Big Data, Ethics, and Society has produced a number of case studies focused specifically on big data research and a white paper with recommendations to start these important conversations (http://bdes.datasociety.net/output/).

Engage your colleagues and students about ethical practice for big data research.

## 7. Develop a code of conduct for your organization, research community, or industry

The process of debating tough choices inserts ethics directly into the workflow of research, making “faking ethics” as unacceptable as faking data or results. Internalizing these debates, rather than treating them as an afterthought or a problem to outsource, is key for successful research, particularly when using trace data produced by people. This is relevant for all research including those within industry who have privileged access to the data streams of digital daily life. Public attention to the ethical use of these data should not be avoided; after all, these datasets are based on an infrastructure that billions of people are using to live their lives, and there is a compelling public interest that research is done responsibly.

One of the best ways to cement this in daily practice is to develop codes of conduct for use in your organization or research community and for inclusion in formal education and ongoing training. The codes can provide guidance in peer review of publications and in funding consideration. In practice, a highly visible case of unethical research brings problems to an entire field, not just to those directly involved. Moreover, designing codes of conduct makes researchers more successful. Issues that might otherwise be ignored until they blow up—e.g., Are we abiding by the terms of service or users’ expectations? Does the general public consider our research “creepy”? [13]—can be addressed thoughtfully rather than in a scramble for damage control. This is particularly relevant to public-facing private businesses interested in avoiding potentially unfavorable attention.

An additional and longer-term advantage of developing codes of conduct is that it is clear that change is coming to big data research. The NSF funded the Council for Big Data, Ethics, and Society as a means of getting in front of a developing issue and pending regulatory changes within federal rules for the protection of human subjects that are currently under review [1]. Actively developing rules for responsible big data research within a research community is a key way researchers can join this ongoing process.

Establish appropriate codes of ethical conduct within your community. Make industry researchers and representatives of affected communities active contributors to this process.

## 8. Design your data and systems for auditability

Although codes of conduct will vary depending on the topic and research community, a particularly important element is designing data and systems for auditability. Responsible internal auditing processes flow easily into audit systems and also keep track of factors that might contribute to problematic outcomes. Developing automated testing processes for assessing problematic outcomes and mechanisms for auditing other's work during review processes can help strengthen research as a whole. The goal of auditability is to clearly document when decisions are made and, if necessary, backtrack to an earlier dataset and address the issue at the root (e.g., if strategies for anonymizing data are compromised).

Designing for auditability also brings direct benefits to researchers by providing a mechanism for double-checking work and forcing oneself to be explicit about decisions, increasing understandability and replicability. For example, many types of social media and other trace data are unstructured, and answers to even basic questions such as network ties, location, and randomness depend on the steps taken to collect and collate data. Systems of auditability clarify how different datasets (and the subsequent analysis) differ from each other, aiding understanding and creating better research.

Plan for and welcome audits of your big data practices.

## 9. Engage with the broader consequences of data and analysis practices

It is also important for responsible big data researchers to think beyond the traditional metrics of success in business and the academy. For example, the energy demands for digital daily life, a key source of big data for social science research, are significant in this era of climate change [36]. How might big data research lessen the environmental impact of data analytics work? For example, should researchers take the lead in asking cloud storage providers and data processing centers to shift to sustainable and renewable energy sources? As important and publicly visible users of the cloud, big data researchers collectively represent an interest group that could rally behind such a call for change.

The pursuit of citations, reputation, or money is a key incentive for pushing research forward, but it can also result in unintended and undesirable outcomes. In contrast, we might ask to what extent is a research project focused on enhancing the public good or the underserved of society? Are questions about equity or promoting other public values being addressed in one’s data streams, or is a big data focus rendering them invisible or irrelevant to your analysis [37]? How can increasingly vulnerable yet fundamentally important public resources—such as state-mandated cancer registries—be protected? How might research aid or inhibit different business and political actors? While all big data research need not take up social and cultural questions, a fundamental aim of research goes beyond understanding the world to considering ways to improve it.

Recognize that doing big data research has societal-wide effects.

## 10. Know when to break these rules

The final (and counterintuitive) rule is the charge to recognize when it is appropriate to stray from these rules. For example, in times of natural disaster or a public health emergency, it may be important to temporarily put aside questions of individual privacy in order to serve a larger public good. Likewise, the use of genetic or other biological data collected without informed consent might be vital in managing an emerging disease epidemic.

Moreover, be sure to review the regulatory expectations and legal demands associated with protection of privacy within your dataset. After all, this is an exceedingly slippery slope, so before following this rule (to break others), be cautious that the “emergency” is not simply a convenient justification. The best way to ensure this is to build experience in engaging in the tough debates (Rule 6), constructing codes of conduct (Rule 7), and developing systems for auditing (Rule 8). The more mature the community of researchers is about their processes, checks, and balances, the better equipped it is to assess when breaking the rules is acceptable. It may very well be that you do not come to a final clear set of practices. After all, just as privacy is not binary (Rule 2), neither is responsible research. Ethics is often about finding a good or better, but not perfect, answer, and it is important to ask (and try to answer) the challenging questions. Only through this engagement can a culture of responsible big data research emerge.

Understand that responsible big data research depends on more than meeting checklists.

## Conclusion

The goal of this set of ten rules is to help researchers do better work and ultimately become more successful while avoiding larger complications, including public mistrust. To achieve this, however, scholars must shift from a mindset that is rigorous when focused on techniques and methodology and naïve when it comes to ethics. Statements to the effect that “Data is [sic] already public” [38] are unjustified simplifications of much more complex data ecosystems embedded in even more complex and contingent social practices. Data are people, and to maintain a rigorously naïve definition to the contrary [18] will end up harming research efforts in the long run as pushback comes from the people whose actions and utterances are subject to analysis.

In short, responsible big data research is not about preventing research but making sure that the work is sound, accurate, and maximizes the good while minimizing harm. The problems and choices researchers face are real, complex, and challenging and so too must be our response. We must treat big data research with the respect that it deserves and recognize that unethical research undermines the production of knowledge. Fantastic opportunities to better understand society and our world exist, but with these opportunities also come the responsibility to consider the ethics of our choices in the everyday practices and actions of our research. The Council for Big Data, Ethics, and Society (http://bdes.datasociety.net/) provides an initial set of case studies, papers, and even ten simple rules for guiding this process; it is now incumbent on you to use and improve these in your research.
